# Medical homelessness and candidacy: women transiting between prison and community health care

**DOI:** 10.1186/s12939-017-0627-6

**Published:** 2017-07-20

**Authors:** Penelope Abbott, Parker Magin, Joyce Davison, Wendy Hu

**Affiliations:** 1 Department of General Practice, Western Sydney University, Locked Bag 1797, Penrith, 2751 Australia; 2 Discipline of General Practice, University of Newcastle, Newbolds Bldg, University Drive, Newcastle, 2308 Australia; 3 Medical Education Unit, Western Sydney University, Locked Bag 1797, Penrith, 2751 Australia

**Keywords:** Prisoner, Health care access, Health services accessibility, Substance misuse, Primary health care, Stigma, Candidacy, Prescription drug misuse, Qualitative research (3–10 keywords)

## Abstract

**Background:**

Women in contact with the prison system have high health needs. Short periods in prison and serial incarcerations are common. Examination of their experiences of health care both in prison and in the community may assist in better supporting their wellbeing and, ultimately, decrease their risk of returning to prison.

**Methods:**

We interviewed women in prisons in Sydney, Australia, using pre-release and post-release interviews. We undertook thematic analysis of the combined interviews, considering them as continuing narratives of their healthcare experiences. We further reviewed the findings using the theoretical lens of candidacy to generate additional insights on healthcare access.

**Results:**

Sixty-nine interviews were conducted with 40 women pre-release and 29 of these post-release. Most had histories of substance misuse. Women saw prison as an opportunity to address neglected health problems, but long waiting lists impeded healthcare delivery. Both in prison and in the community, the dual stigmas of substance misuse and being a prisoner could lead to provider judgements that their claims to care were not legitimate. They feared they would be blocked from care even if seriously ill. Family support, self-efficacy, assertiveness, overcoming substance misuse, compliance with health system rules and transitional care programs increased their personal capacity to access health care.

**Conclusions:**

For women in transition between prison and community, healthcare access could be experienced as ‘medical homelessness’ in which women felt caught in a perpetual state of waiting and exclusion during cycles of prison- and community-based care. Their healthcare experiences were characterized by ineffectual attempts to access care, transient relationships with healthcare providers, disrupted medical management and a fear that stigma would prevent candidacy to health care even in the event of serious illness. Consideration of the vulnerabilities and likely points of exclusion for women in contact with the criminal justice system will assist in increasing healthcare access for this marginalised population.

## Background

Women in prison have poor self-reported health and high levels of social disadvantage, experience of trauma and mental health problems [1–4]. In Australia, women are usually in prison for less than 6 months, re-incarceration is common and the majority report problematic substance misuse [3–6].

Approximately 8% of people in prison in Australia are women and the imprisonment rate for women is increasing, currently standing at 33 prisoners per 100,000 female adult population [7]. Aboriginal and Torres Strait Islander women are over represented in prison, related to historical and systemic disadvantage, and these women are even more likely to experience serial incarcerations with short sentences or on remand [5].

Poor access to health care is common for women in contact with the criminal justice system. Substance misuse and struggles related to accommodation, socioeconomic disadvantage and family needs can mean health is neglected in the community [8, 9]. In a national survey of people in prison in Australia in 2015 [10], 48% of women reported they did not access the health care they needed when they were in the community. Additionally 15% said they did not access needed care in prison. The main reason reported for men and women not accessing care in either setting were reported to relate to choosing not to and lacking motivation to seek care. Additional barriers in the community were reported to be cost, substance misuse and competing priorities, while in prison, waiting times and health care not being available when needed were the other major barriers.

Although prison is often a time of compromised wellbeing due to the deprivation and loss of choice and control inherent to incarceration [11], it can also be a window of opportunity to improve health through access to overdue health care [12, 13]. Furthermore, the importance of managing health well across the interface of prison and community is clear. Leaving prison is a time of vulnerability, associated with high morbidity and mortality [14–16]. Health problems at release decrease the likelihood of successful community re-entry [15]. However, the ideal of post-release continuity of care can be disrupted by complex health and social support needs, relapse to substance misuse, poor health information transfer and difficulty in establishing connections with community healthcare providers [17–19].

In this study, we examined the ways in which women in contact with the prison system experience access to health care, particularly those with histories of problematic substance misuse. We focused on women who were exiting prison and aiming to re-establish their lives in the community, and explored their experiences of both prison and community health systems. Through understanding their experiences of healthcare access, healthcare providers and health services may be better enabled to provide equitable care for this marginalised group.

### Theoretical framework

We used the conceptual framework of candidacy as described by Dixon-Woods and colleagues [20] to examine the women’s healthcare access. The framework was first developed to examine equity of access to the United Kingdom National Health Service, thus providing a useful lens on how access is determined and enabled for people in disadvantaged situations. It emphasizes that healthcare access is contingent and subject to constant negotiation. Candidacy has been applied to healthcare access in diverse situations including people with intellectual disability [21], mental health problems [22], multiple sclerosis [23], young people seeking sexual health care [24], women who were sex workers needing primary care [25] and children with asthma [26]. It has not yet been applied to people in contact with the criminal justice system and people with histories of substance misuse in the research literature.

As explained by the candidacy framework, potential service users identify a health need and seek care (labelled ‘*identifying*’ and ‘*appearing*’). After care has been requested, providers are seen as ‘*adjudicating’* the claims, deciding whether and in what way care will be delivered. Providers’ judgements can be based on how deserving potential service users are and how well they will do if given treatment, which can disadvantage those in more deprived circumstances [20]. Limited resources, such as in prisons and hospitals, may increase adjudications of ineligibility by raising thresholds for what is thought to be a legitimate need. The candidacy framework also considers the *‘navigation’* and *‘permeability’* of services. To navigate services, potential users must be aware of them and have adequate resources such as transport and time. Permeability refers to the ease with which people can use services, including through feeling comfortable and having the capabilities to access the service. For example, services which align with user cultural values are more permeable an﻿d servi﻿ces﻿ with complex or rigid referral and appointment systems are less permeable.

## Methods

Given the ethical and practical challenges of recruiting people in prison as research participants, we report our methods in detail according to the Standards of Reporting Qualitative Research guidelines [27]. The principal researcher (PA), who undertook all interviews, was employed as a part-time general practitioner (GP) in the prison health service and also worked as a GP in the community.

### Setting

This study took place in 3 women-only correctional centres in New South Wales (NSW) Australia. Health care for women in NSW prisons is delivered predominantly in state-owned correctional centres through a Board-governed network under the NSW Ministry of Health [28]. Health care is primarily delivered by general and specialist nurses [6, 29]. Women see GPs and other medical practitioners after being triaged by nurses to waiting lists. This differs from the community model, where GPs provide most primary health care and are directly accessible under universal health insurance.

### Sampling and data collection

We invited women who were within 6 weeks of release to participate in two interviews, firstly in prison and then 1–6 months after release. Women were eligible if they had been in prison at least 1 month, could be interviewed in English without an interpreter and if they had not received health care from PA beyond treatment for minor self-limited problems.

We identified potential participants through self-response to flyers, custodial lists and nursing and correctional staff knowledge of pending release dates. Women were invited by staff to meet with the researcher. Initially all eligible participants who responded to flyers were recruited. To ensure maximum variation, nursing staff subsequently identified participants who varied in age, ethnicity, custodial history, health status, healthcare utilisation and engagement in transitional support programs [30]. PA undertook the consent process with all participants, emphasising the voluntary and confidential nature of the research and that decisions to participate would have no effect on their health care or relationships with healthcare providers.

Interviews in prison were conducted in prison health clinics or general visitor areas under general surveillance of correctional officers outside the interview rooms. Post-release interviews in the community were by telephone. Participants received a payment of $10 AUD into their in-prison account consistent with usual research practice in NSW prisons, or a $50 AUD supermarket voucher, if in the community.

Interviews were semi-structured and questions explored needs, expectations and experiences of health care with participant-led content encouraged. Focused questions were added to explore themes identified in the emerging analysis [31]. Interviews were audiotaped and transcribed verbatim.

### Data analysis

Given that many participants spoke of experiences in multiple incarcerations, we analyzed women’s pre and post-release interviews together as continuing narratives of their experiences of health care. We used inductive thematic analysis informed by constructivist grounded theory [31]. The constructivist approach was considered appropriate for this research as it is encourages recognition of, and ongoing reflection on, how researcher perspectives, position and privilege influence the analysis. PA undertook open coding on all transcripts concurrently with data collection. WH and JD independently coded a third of selected information-rich transcripts to enhance rigor and JD also provided interpretations arising from her Aboriginal cultural expertise. Focused coding and analysis proceeded with repeated reference back to the data, memo-writing, checking of the emerging analysis in new interviews with participants and research team discussions. We further reviewed the findings using the theoretical lens of candidacy to generate additional insights on healthcare access.

## Results

**Fig. 1:**
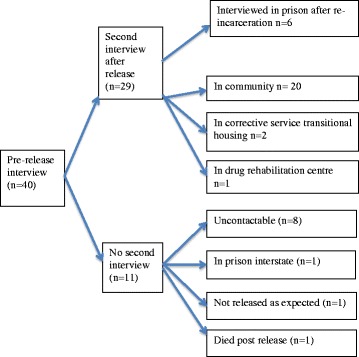
Participant outcomes

We interviewed 40 women prior to release and 29 of these women in a second interview. Their characteristics are described in Table 1. The majority of women had problematic substance misuse (35/40). The average duration of pre-release interviews was 28 min and second interviews, 22 min. The location of interviews and reasons for not participating in a second interview are shown in Fig. 1. Seven women returned to prison within 6 months of release. One woman died of an overdose.

**Table 1:** Description of participants (*n* = 40)

Characteristics	n
Age	
19–24	6
25–30	11
31–40	13
41–50	6
51–59	4
Background	
Aboriginal	16
Torres Strait Islander	1
Culturally and linguistically diverse^a^	6
Self-identified health issue of concern	
Substance misuse	35
Mental health	31
Physical health	25
Nil	2
Affiliated with formal release support program	
Transitional support by prison-based health linkage programs^b^	7
Residential transitional program	2
Residential drug rehabilitation	2
Community-based case management program	2
Custodial details	
Previous incarceration	
Yes	25
No	15
Incarceration duration	
Less than 3 months	4
3 < 6 months	6
6 < 12 months	21
12–24 months	9

^a^Defined as speaking a language other than English at home

^b^Prison-community linkage programs in place prior to release. For 5 of 7 women case management continued for 1 month after release

Due to commonality of experiences across prison and in the community, findings from both settings are presented together. Women’s experiences pertained largely to primary health care delivered by prison-based nurses and doctors and by community GPs, but also to hospital-based providers including Emergency Departments. The major themes related to the opportunity to access health care in prison and the constraints in that environment; being seen as legitimate seekers of care; the experience and fear of being blocked from care; and the services and personal capabilities which promoted access to care. These are explored below with illustrative quotes.

### Prison as a health care opportunity

Despite the many disadvantages of being in prison, women also believed it to be an opportunity to seek overdue care for preventive health and neglected health problems. Although good health was seen as desirable in the community, it could be difficult to achieve. Increased focus on health in prison was possible because of decreased substance misuse, mental health treatment, time on their hands, fewer competing priorities and a desire to make positive life changes.

*When you come in here, is when you really are straight and you really want to know if you’ve got anything … your head becomes clearer and then you do think about your health as you’re getting older. (Participant 4).*

Some women moved in and out of prison so frequently that they saw prison health services as their main provider.

*The only time I – I literally see doctors and that is in gaol… I’m not out long enough to get that appointment. (Participant 30).*

For some, health care in prison was better aligned with their needs than care they had experienced in the community due to prison clinicians’ understanding of addiction and its comorbidities. Women believed community GPs lacked interest and skills in substance misuse management and therefore women were more likely to disclose and seek care for this in prison. Hepatitis C treatment in prison was often mentioned as a healthcare opportunity and one which could create personal meaning out of being in prison.

*I wanted to take something positive out of this experience, ‘cause it’s been an ordeal - … to address whatever I could to make the most of this time rather than to have it dead time. (Participant17).*

### Constraints in prison care - ‘the waiting game’

However, prison could also be experienced as a missed opportunity. The key systemic constraint was long and unpredictable waits for care. Several women referred to this as ‘the waiting game’. Preventive health care delivered by nurses was effective and valued, but if women required access to a GP, secondary care or specialized investigations, waiting times could be substantial. Some women saw the waits as acceptable because care was ultimately delivered, particularly during longer sentences. Other women strongly felt waiting put them at risk of health complications, and waiting could be interpreted as a judgement that their problems weren’t important, or as withholding of care. Their frustration was magnified by wanting to have care completed while in prison, as they believed they would not follow up in the community. Women with shorter sentences reported deflection of health care requests because investigations or specialist care could not realistically be achieved before release. Some women did not seek care while in prison because of previous experiences of waiting.

*I’m like, “Well I’m going home soon. Within a week or two I’m going home and now you see me.” … It would have been so much easier than out there. Like, my life’s full-on out there. (Participant 8).*

Another constraint was the limited range of care compared to the community. Usual medications, alternative therapies, dietary preferences and preferred healthcare options were not always available.

*You’ve got more options out there. You’ve got counsellors, um, you’ve got groups that you can go to. (Participant 32).*

### Legitimacy and stigma in prison and the community

Women perceived they were frequently judged not to have health problems worthy of receiving care and were denied health care both in prison and in the community. They described this as a battle to be seen as legitimate patients and experienced this as personal rejection, linked to the dual stigmas of substance misuse and imprisonment.

*The drug user could be having a leg hanging off and [the community GP thinks]‘Oh well. She just got released from gaol. She’s – she looks like a user, so couldn’t harm her to wait another 10 min, 5 minutes, whatever. I’ll just see this family’. (Participant 30).*

*You’re not trying to get pills, you know, because you want them. It’s when you’re a genuine person and, you know, you - you think there is something wrong with you, you’d like to feel safe and feel like [prison healthcare providers] are there for you, and I just don’t think they have been. (Participant 9).*

Being refused care at GP practices in the community could be experienced as a profound and traumatizing rejection. This could occur because of past behaviours leading to permanent barring from practices, or when GPs suspected prescription drug misuse. Some women believed that their requests for mental health care were misinterpreted by community GPs as drug seeking due to stigma and lack of GP skills. Waiting room signs aimed at deterring prescription drug misuse could reinforce perceptions of lower status and women reported a heightened sensitivity to the inclusion of past medical opinions in their health records.

*[Community GPs] treat you like, you know, you’re nobody really… It has to be something in my file that someone’s put in there that, straightaway, discriminating against me. (Participant 13).*

Participants who did not have a history of substance misuse perceived prison healthcare providers to be accustomed to managing women with addictions, and the system to be set up accordingly, such that they also experienced lack of credibility in their claims to care. While their access to community providers was satisfactory, in prison they felt a need to differentiate themselves from other prisoners with substance misuse histories. At times this appeared to relate to their own negative attitudes to addiction. They reported that women with substance misuse problems took excessive healthcare provider attention, with providers disbelieving their own, more legitimate claims to care.

*The ones that are not druggies, they’re the ones that really need help. (Participant 39).*

Some women felt that healthcare providers both in and outside the prison didn’t believe them when they discussed their medical histories, and particularly their reported medications, requiring ‘proof’ before instituting treatment. They considered this to be emblematic of their ongoing struggle to be seen as ‘legit’. One participant expected community GPs to be suspicious of any information she gave them, even official paper-based test results which needed follow up.

*Maybe they’ll think [the test result] it’s not legit or something… They would think it was fake… because it’s got to do with prisons and criminals. (Participant 7).*

With such experiences over time, some women chose not to seek care in prison or the community because they assumed providers would not be receptive, or the care they would receive would be substandard. In the community, women could choose not to disclose their incarceration to avoid differential treatment.

*The doctors outside don’t know that you’ve been to gaol. You don’t have to tell them anything, you know what I mean. So there’s no real stigma when you’re out. (Participant 11).*

Conversely, access was facilitated by having a health condition which was prioritized by healthcare providers, such as HIV or schizophrenia. When seeking healthcare access these otherwise stigmatising conditions could reinforce women’s status as legitimate patients both in prison and the community, increasing their ability to access services and receive continuity of care.

Some health services were considered inclusive of people with histories of substance misuse or incarceration, such as sexual health services and services which catered for marginalised members of the community. In Aboriginal Medical Services, women reported there was usually no stigma related to their status as ex-prisoners, however substance misuse could still be a source of stigma.

*[I go to] Aboriginal medical centres ‘cause not many discriminate I don’t think. I don’t know. Well there’s some do I reckon and some don’t really. When you say you’re a drug user and they blurt “huh,” you know what I mean? (Participant 33).*

Despite anger at not being seen as legitimate when they believed care was needed, some women also acknowledged the complexity of prescription drug misuse, the danger this posed to them, and the prescriber’s role in accurately judging the legitimacy of requests for medications.

*You get the doctor to write it for you anyway, which is not the doctor’s fault. It’s the person’s fault for lying. (Participant 4).*

### Being let down and blocked from care

Women related experiences of feeling uncared for and let down by providers in prison and in the community. Women commonly reported not being called up to the prison clinic or contacted by community providers despite their attempts to seek care, interpreting this as withholding of care and a judgement they were not important.

*I want to be treated like a normal patient, you know, that wants to get something done… It’s just gaol, it makes you feel like a number, you know. But, um, yeah, I guess, when you get out, you just, yeah, no-one really – no-one cares for when you get out. (Participant 7).*

Differential treatment was seen to have serious implications. Women feared the possibility of being blocked from care despite a serious health problem, fearing misdiagnosis, uncontrolled pain or life threatening illness. This was seen as a risk both when in prison, for accessing hospital emergency departments whilst a prisoner, and when accessing GPs and hospitals in the community.

*I said, “Oh, no I don’t use drugs anymore,” but what [the community GP] wrote was reflecting on me as a drug-user, and I was treated differently. Yeah. Especially when I went to hospital for my gallstones, one time, they wouldn’t medicate me because they thought I was a morphine seeker… I wouldn’t even know how to seek morphine. (Participant 8).*

### Capabilities, self-efficacy and supporting access

Capabilities for accessing both prison and community-based health systems related to family support, self-efficacy, assertiveness and knowledge of and compliance with the rules of different systems. Those who did not successfully meet formal requirements, for example by carrying their medical benefits cards or attending appointments, were likely to conflict with providers. Some women described being vocal and determined in seeking care, changing providers when necessary until they received the care they needed.

*I had to change doctors because I was refused by two doctors in [name of town] when I got out of custody the last time… I have an alcohol problem - and I missed appointments… I think [the barring doctor] must’ve thought I was looking for drugs, pain medication or, you know, making it up. But I most certainly wasn’t making it up. (Participant 3).*

Women who lacked confidence in their ability to manage their health often invoked their previous lack of success. Mental health problems, addiction, social isolation and poor life experiences and circumstances decreased their sense of self-efficacy. Self-efficacy was reported to be increased by existing personality traits and resilience, personal growth and overcoming addiction.

*If I can’t look after myself, who’s going to look after me? … I’ve always known how to get help. (Participant 2).*

Some believed the passive role they assumed in prison decreased their confidence in accessing care after release. Others reported increased self-confidence at release related to overcoming pre-incarceration health problems or to positive healthcare experiences while in prison. Healthcare providers could be important in supporting women’s self-confidence.

*I’ve addressed more issues since coming to gaol than I ever did … I’ve taken a good look at all that has affected me in my life so it’s been quite a positive experience coming to gaol… I can identify what’s going on and I can get myself help. (Participant 40).*

*When you hear a good thing said about you by a doctor or a nurse … it really means a lot, like, you know that it’s true and, like, if they think that, then – you know what I mean? And it gives you a bit of confidence and a bit of strength. (Participant 21).*

Transitional programs, care coordinators or mentors were seen to be effective facilitators to care on leaving prison. They were valued for practical and emotional support particularly for women who had little family support. Linkages with community healthcare providers were also enhanced by transitional case managers who also acted as advocates and communication brokers.

*If you’re unsure, and if you’re not very good at speaking or whatever, like, to go to the doctors or communicating - or anywhere that you need to go, [the care coordinators], you know, they’ll help you with that. (Participant 22).*

## Discussion

Women in our study experienced significant barriers to healthcare access both in prison and in the community, particularly related to their histories of substance misuse. Many sensed that they were not perceived to be legitimate patients with legitimate healthcare needs, which created a fear of being blocked from care when it was urgently needed.

### Candidacy for health care

The candidacy framework can be used to uncover vulnerabilities in access [20]. In our study of women in contact with the criminal justice system, concepts related to making claims to care (*identifying* and *appearing*) and judging of eligibility by providers (*adjudication)* were illuminating.

#### Claims to care

Dixon-Woods and colleagues note that marginalised groups may be more likely to identify themselves as candidates for care through a series of crises rather than planned health care, resulting in high uptake of emergency care compared to preventive care [20]. This accords with findings from a large survey of Australian prisoners in 2009, who reported high uptake of hospital emergency department care in the community [3, 4].

The increased help-seeking behaviour seen in prison [10] has been suggested as linked to increased distress caused by incarceration [32]. However, in our study, the main motivator for seeking care was greater self-identification of candidacy due to decreased substance misuse, fewer competing priorities and a desire for positive life change. Women wanted to address overdue healthcare needs. Prison was seen as a healthcare opportunity, however one which could be missed due to system constraints.

In our research, prison health services were seen to perform well in providing preventive health care but were less able to deliver complete investigation or management of more complex health needs within the confines of a prison sentence. In prison, care is delivered within a correctional system which is ill-designed for healthcare delivery. There are time-limited windows of access within a regulated daily schedule, and a transient prison population serving sentences which may be short or include frequent movements between prisons [6]. After women *identified* a healthcare need and *appeared* to the prison health service, the rest of the prison sentence could be spent waiting for the health management plans made in those consultations to be implemented. Waiting had a negative effect on relationships with prison healthcare providers and could be interpreted as providers withholding care or judging women’s claims as unimportant.

#### Relationships with healthcare providers

Women describe a struggle to establish their legitimate access to care both in prison and in the community because of negative provider *adjudications*. Prescription drug misuse affects therapeutic relationships both in prison and the community. Prison doctors perceive one of their key tasks is judging patient credibility [32] and the challenge in being considered a legitimate patient in prison has been described [33, 34]. In the community, stigma is compounded by healthcare provider discomfort and lack of skills in managing ex-prisoners or substance misuse problems [18, 35], which the women in our study readily identified.

Mental illness is a known source of stigma within primary care which can hinder help seeking [36]. In our study, the stigma of mental illness was not seen to impede healthcare access. Rather, women perceived their mental health care was suboptimal because they were not taken seriously by providers who suspected exaggeration related to their addictions.

Provider adjudication had a profound emotional meaning for many women in this study, imbued with expectations and experiences of rejection and withholding of care. In other studies of access using candidacy theory, service users could feel devalued by negative interactions with providers [23] and frustrated by delays in diagnoses [26] or ineligibility for programs [21]. However the fear of being denied future care for serious illness illustrates the heightened significance of provider adjudications to women with substance misuse and in contact with the criminal justice system.

Overcoming stigma may require women to be articulate and persistent both in and out of prison, consistent with the candidacy concept that negotiation between providers and users is a key factor in accessing care. The power imbalance between providers and patients can make negotiations challenging for patients in many healthcare situations, but even more so for prisoners, who have controls and limits on their choices in prison. Although prisons may aim to release more empowered individuals with control over their lives, agency may decrease in prison and persist after release, as part of the institutionalization that can be fostered by serial incarcerations [37].

### Experiencing ‘medical homelessness’

A key aim of primary care is to reduce health inequalities by providing coordinated whole-person care, also an underlying principle behind the recent emergence of patient-centered medical homes [38]. However, in the same way that women’s lives are destabilized by lack of accommodation on leaving prison [39], our research also shows that they are destabilized by a lack of access to trusted and reliable medical care. Furthermore, women can be caught in an ongoing state of waiting and exclusion during cycles of prison and community-based health care, leading to a persistent state of transition and ‘medical homelessness’.

Their medical homelessness is characterized by ineffectual attempts to access care, transient relationships with healthcare providers, disrupted medical management and a profound sense of exclusion from health care. Health system constraints, provider judgements that their claims to care are not legitimate and experiences of poor provider skills in managing addiction and its comorbidities contribute to a sense that they have no place in either prison or community-based health care. Experiences of rejection contributed to an ongoing state of inadequate care by engendering avoidance and helplessness in our participants.

At a practical level, women in contact with the prison system are a transient population. Women may frequently move between prison and community on multiple short sentences, a particular problem for Aboriginal and Torres Strait Islander women. Custodial decisions may lead to them being placed in different prisons or in unfamiliar community locations on release. Developing trusting therapeutic relationships with providers when displaced from familiar settings is difficult, and even more so if the basis for trust is eroded by providers who assume drug seeking, regardless of the presenting health problem.

Although control in the prison environment led to some women being more able to seek care, their custodial situation also created barriers which meant women could leave prison feeling their needs were not met. Women on remand are not eligible for all prison-based health programs and not all services available in the community are accessible in prison. If health care is not completed prior to release, initial efforts may be wasted by failures of continuity due to disconnected systems of care [9, 19] or by choices to not to disclose incarceration after release [18].

Women who have been in contact with the criminal justice system have often had poor life experiences including trauma, abuse and violence. Our participants’ sense of personal rejection and of falling between the cracks of health care are likely to be based both on experienced events as well as on psychological vulnerability related to life trauma and experiences of being let down throughout their lives. Their deep and often lifelong disadvantage is perpetuated in the personal and structural barriers they face in accessing health care both in prison and the community.

### Overcoming barriers to care

Skilled and empathic healthcare providers assist in overcoming barriers to care. Women in contact with the prison system value community GP acknowledgement of, and assistance with, the broad issues that have an impact on their wellbeing, as well as skilled management of substance misuse and a non-judgemental patient-centred approach [18]. Exposure of students and trainees to people in prison or with substance misuse problems may decrease stigma and promote more effective health care for these people [40, 41]. This should include training in trauma-informed health care so that healthcare providers are aware of the psychological dynamics that may impact on the development of therapeutic relationships with people in contact with the custodial system [42]. This may assist providers to avoid re-traumatizing vulnerable patients, for example through words and actions which reinforce the sense of withholding care.

Family and other advocates can greatly assist access to care [21]. However, women leaving prison often lack social connectedness and support in the community [43]. Access may be facilitated by prison and community providers working together prior to women leaving prison to plan for care following release [44]. Care navigation through re-entry programs can provide instrumental and relational support to promote health care access [9, 30, 45]. Given the risk of medical homelessness, our study reinforces the importance of resourcing transitional programs to assist women to link with skilled, non-judgmental community care on release.

### Limitations

The participants in this study had high reported health problems and needs particularly related to substance misuse. Although our participants also reported mental and physical health problems, their primary focus when reporting barriers to healthcare access revolved around current or past histories of substance misuse. Our findings are likely to be more transferable to other people who struggle with substance misuse, both inside and outside prisons. Although our participants were reflecting on their experiences as women within the Australian prison and community health system, the applicability of candidacy concepts suggests wider relevance for marginalised groups, particularly those caught in a pattern of serial incarcerations or of substance misuse.

The roles of the primary researcher and interviewer as a visiting GP within the prison health service and as a community GP were made known to the participants. Although this may have enhanced the research through shared understandings of complex health systems, it may also have inhibited participants from expressing their views completely, and led to lack of identification of findings which may be novel to an outsider. However, the fact that the women freely shared in their experiences of suboptimal care suggests that they did not feel constrained by a fear of further impacting on their access to care. The inclusion of researchers who are not involved in delivery of prison health services and a cultural adviser assisted in ensuring the analysis was comprehensive and inclusive of multiple perspectives and interpretations.

## Conclusion

Women in contact with the criminal justice system, and particularly those with histories of substance misuse, can face difficulties in accessing health care both in prison and in the community. For those women who cycle in and out of prison, healthcare access can be conceived as an ongoing state of ‘medical homelessness’. Their experiences of poor community provider skills in managing addiction and provider judgements, both in prison and in the community, that their claims to care are not credible may contribute to a persistent state of waiting and exclusion during cycles of prison and community-based care. Consideration of the vulnerabilities and points of exclusion for women caught in this cycle will assist in determining how to ensure healthcare access for this marginalised population.
